# Directly induced human Schwann cell precursors as a valuable source of Schwann cells

**DOI:** 10.1186/s13287-020-01772-x

**Published:** 2020-06-26

**Authors:** Han-Seop Kim, Jae Yun Kim, Cho Lok Song, Ji Eun Jeong, Yee Sook Cho

**Affiliations:** 1 Stem Cell Research Laboratory (SCRL), Immunotherapy Research Center (IRC), Korea Research Institute of Bioscience and Biotechnology (KRIBB), 125 Gwahak-ro, Yuseong-gu, Daejeon, 34141 South Korea; 2 Department of Bioscience, KRIBB School, University of Science & Technology, 113 Gwahak-ro, Yuseong-gu, Daejeon, 34113 South Korea

**Keywords:** Direct reprogramming, Schwann cell precursors, Schwann cells, Peripheral nerve system, Nerve repair

## Abstract

**Background:**

Schwann cells (SCs) are primarily responsible for regeneration and repair of the peripheral nervous system (PNS). Renewable and lineage-restricted SC precursors (SCPs) are considered highly desirable and promising cell sources for the production of SCs and for studies of SC lineage development, but SCPs are extremely limited. Here, we present a novel direct conversion strategy for the generation of human SCPs, capable of differentiating into functional SCs.

**Methods:**

Easily accessible human skin fibroblast cells were directly induced into integration-free SCPs using episomal vectors (Oct3/4, Klf4, Sox2, L-Myc, Lin28 and p53 shRNA) under SCP lineage-specific chemically defined medium conditions. Induced SCPs (iSCPs) were further examined for their ability to differentiate into SCs. The identification and functionality of iSCPs and iSCP-differentiated SCs (iSCs) were confirmed according to morphology, lineage-specific markers, neurotropic factor secretion, and/or standard functional assays.

**Results:**

Highly pure, Sox 10-positive of iSCPs (more than 95% purity) were generated from human skin fibroblasts within 3 weeks. Established iSCPs could be propagated in vitro while maintaining their SCP identity. Within 1 week, iSCPs could efficiently differentiate into SCs (more than 95% purity). The iSCs were capable of secreting various neurotrophic factors such as GDNF, NGF, BDNF, and NT-3. The in vitro myelinogenic potential of iSCs was assessed by myelinating cocultures using mouse dorsal root ganglion (DRG) neurons or human induced pluripotent stem cell (iPSC)-derived sensory neurons (HSNs). Furthermore, iSC transplantation promoted sciatic nerve repair and improved behavioral recovery in a mouse model of sciatic nerve crush injury in vivo.

**Conclusions:**

We report a robust method for the generation of human iSCPs/iSCs that might serve as a promising cellular source for various regenerative biomedical research and applications, such as cell therapy and drug discovery, especially for the treatment of PNS injury and disorders.

## Background

Schwann cells (SCs), the main glial cells of the peripheral nervous system (PNS), are developmentally derived from neural crest cells (NCCs) via intermediate Schwann cell precursors (SCPs) [1]. Autologous human SCs isolated from adult peripheral nerve biopsies with minimal risk of host immune rejection have been suggested for the treatment of human peripheral nerve injury [2, 3], but insufficient donor materials are still a major hurdle in achieving robust clinical improvement. Recently, advanced stem cell technologies, such as lineage-specific differentiation and reprogramming, have offered a better opportunity to obtain engineered therapeutic human SCs from various other types of accessible cell sources.

Human pluripotent [embryonic stem cells (ESCs) and induced pluripotent stem cells (iPSCs)] [4, 5] and multipotent stem cells from adipose [6, 7], bone marrow [7], umbilical cord [8], dental pulp [9, 10], epidermal keratinocytes [11], and muscle [12], which have NCC differentiation/trans-differentiation capacity, and are capable of differentiating into SCs, have been demonstrated as alternative sources of SCs. To date, human pluripotent stem cells (hPSCs) with high expansion and differentiation capacity are considered the most ideal renewable sources to generate high numbers of SCs through an intermediary NCC stage, but their wider uses are still limited by long differentiation time and low functionality [4, 5, 13–16]. Although great progress has been made in nerve tissue engineering and biomaterials to aid the process of nerve regeneration, the use of differentiated SCs to support neurons and form myelin sheaths in vitro and in vivo is still unsatisfactory, especially for long-gap nerve injury [13, 17–19].

Recently, a direct reprogramming strategy offering a more convenient, less labor-intensive, and faster procedure has emerged as another alternative approach for producing SCs. Overexpression of the transcription factor (TF) Sox10, in combination with extracellular matrix components, epigenetic modifiers, and small molecule activation of WNT, has been shown to directly induce multipotent NCCs from human fibroblasts, which could differentiate into SCs [20]. Notch1 (NIC) overexpression-induced NCCs from melanocytes did not show differentiation potential into SCs [21]. In addition, Sox10, in combination with Egr2 [22] or Krox20 [23], could directly convert human fibroblasts into iSCs without passing through the iPSC state. Sox10/Egr2 and Sox10/Krox20-induced SCs (Sox10/Krox20-iSCs) have been shown to improve in vitro myelination in cocultures with dorsal root ganglion (DRG) while only Sox10/Krox20-iSCs have been shown to be effective in sciatic nerve regeneration in vivo, even at low frequency [22, 23]. Without the use of ectopic transgene overexpression, iSCs could be converted from human fibroblasts using a small molecule-based conversion strategy through passing a transient neural precursor stage [24]. However, the functionality of directly induced SCs on in vitro myelination and in vivo transplantation remains to be further evaluated.

Previously, we have reported a novel differentiation method for hPSCs into SCPs using a chemically defined sequential stage-specific culture medium [25]. In addition to lineage-specific TFs, pluripotency TFs have been widely used for the direct induction of functional cell types from one cell lineage to another. In this study, we aimed to develop an SCP-targeted direct reprogramming protocol with master pluripotency TFs, thereby providing a highly valuable and accessible source of human SCPs/SCs for SC lineage development and a new therapeutic approach.

## Methods

### Animals

C57BL/6 male mice used for the sciatic nerve injury model were purchased from Dae Han BioLink Co., Ltd. (Chungbuk, South Korea) and kept in a conventional animal care facility at Korea Research Institute of Bioscience and Biotechnology (KRIBB). All animal experiments were approved by the Institutional Animal Care and Use Committee of KRIBB (KRIBB-AEC-18041).

### Generation of iSCPs from human fibroblasts

For iSCP generation, 1 × 10^6^ human skin fibroblasts (CRL-2097; ATCC, Manassas, VA) were electroporated with Epi5™ episomal iPSC reprogramming vectors (OCT4, SOX2, KLF4, MYCL1, LIN28 and p53 shRNA, Cat. No. A14703, Thermo Scientific, Waltham, MA) using a Microporator MP-100 (Thermo Scientific, Waltham, MA) and plated on growth factor reduced Matrigel (BD Bioscience, San Jose, CA)-coated culture dishes in fibroblast medium (FM) containing 10% FBS in α-MEM. After 2 days of induction, the culture medium was changed to reprogramming induction medium-I (RIM-I) containing 10% FBS (Thermo Scientific), 5% KnockOut serum replacement (Thermo Scientific), 1% NEAA (Thermo Scientific), 0.11 mM β-mercaptoethanol (Thermo Scientific), 3 μM CT 99021 (Tocris, Bristol, UK), 0.1 mM Na-butyrate (Tocris), 10 ng/ml bFGF (Peprotech), 2 μM panate (Tocris), 0.5 μM RG108 (Tocris), and 0.5 μM NECA (Tocris) in DMEM/F12. On day 7 of culture, the culture medium was switched from RIM-I to RIM-II containing 1xN2 (Thermo Scientific), 1xB27 (Thermo Scientific), 0.005% BSA (Thermo Scientific), 2 mM GlutaMax-I (Thermo Scientific), 0.11 mM β-mercaptoethanol, 3 μM CT 99021, and 20 μM SB431542 (Tocris) in advanced DMEM/F12 and neurobasal medium (1:1 mix). On day 9 of culture, the culture medium was switched from RIM-II to RIM-III containing 1xN2, 1xB27, 0.005% BSA, 2 mM GlutaMax-I, 0.11 mM β-mercaptoethanol, 3 μM CT 99021, 20 μM SB431542 (Tocris), and 100 ng/ml NRG1 (Peprotech, Rocky Hill, NJ) in advanced DMEM/F12 and neurobasal medium (1:1 mix). Each medium was changed every other day. On day 18, SCP colonies were picked and dissociated with accutase (Millipore, Billerica, MA) treatment and expanded by an additional incubation in RIM-III.

### Differentiation of SCPs into SCs

SCs were differentiated as previously described [25]. Briefly, SCPs were cultured on Matrigel-coated plates in SC differentiating medium (SCDM) containing 0.2% FBS (Thermo Scientific), 200 ng/ml NRG1 (Peprotech), 4 μM forskolin (Sigma, St. Louis, MO), and 10 ng/ml PDGF-BB (Thermo Scientific) in DMEM. Two days later, the culture medium was changed with medium containing 0.2% FBS and 100 ng/ml NRG1 in DMEM. The culture medium was replaced every other day.

### Quantitative real-time reverse transcription-polymerase chain reaction (qRT-PCR)

Total RNA was extracted from cells using an RNeasy Mini Kit (Qiagen, Valencia, CA) following the manufacturer’s instructions. Two micrograms of RNA was reverse-transcribed with the SuperScript®VILO™ cDNA Synthesis Kit (Thermo Scientific) according to the manufacturer’s instructions. PCR was performed with SYBR and analyzed using the 7500 Fast Real-Time PCR system (Applied Biosystems, Foster, CA). The primer list is shown in Table S1.

### Immunocytochemistry

Cells were fixed with 4% formaldehyde (Sigma) in PBS for 10 min and then washed with PBS four times for 10 min. The fixed cells were incubated and permeabilized with 0.2% Triton X-100, 10% FBS, and 1% BSA in PBS for 1 h at room temperature. The cells were treated with primary antibody in PBS containing 1% BSA for 1 h at room temperature. The cells were washed with PBS three times and incubated at room temperature for 20 min in PBS containing 1% BSA with anti-mouse Alexa 488-conjugated (1:400, Thermo Scientific), anti-mouse Alexa 546-conjugated (1:400, Thermo Scientific), anti-rabbit Alexa 488-conjugated (1:400, Thermo Scientific), anti-rabbit Alexa 546-conjugated (1:400, Thermo Scientific), or anti-rat Alexa 647-conjugated (1:200, Thermo Scientific) secondary antibodies. Immunocytochemistry images were obtained by using an Axio VertA.1 microscope (Carl Zeiss, Oberkochen, Germany).

The sciatic nerves were obtained from mice and dissociated as previously described [25]. Briefly, the mice were perfused transcardially with 4% paraformaldehyde in PBS. The sciatic nerves were isolated and postfixed in the same solution for 24 h at 4 °C overnight. Fixed sciatic nerves were immersed in 30% sucrose in PBS and then embedded in OCT compound (Sakura Finetek USA Inc., Torrance, CA). Sciatic nerve sections with a thickness of 15 μm were prepared using a Cryostat, and then, frozen sections were stained with the respective antibodies as previously described [25]. The antibodies used are listed in Table S2.

### Flow cytometry

Cells were fixed with 2% formaldehyde (Sigma) in PBS for 10 min and washed 3 times with PBS containing 1 mM EDTA. The fixed cells were blocked and permeabilized with 0.5% Tween-20 (Sigma), 1 mM EDTA, and 0.5% BSA (Millipore) in PBS for 20 min. The cells were incubated with conjugated primary antibody (Table S2) in PBS containing 2% BSA for 10 min at room temperature. Isotype control IgG (Biolegend, San Diego, CA) was used as a negative control for gating. After the antibody reaction, the cells were washed with PBS 2 times and analyzed using BD Accuri C6 (BD Biosciences, Billerica, MA).

### Western blotting

Cell lysates were prepared in RIPA buffer containing 50 mM Tris–HCl, pH 8.0, 150 mM sodium chloride, 1% Triton X-100, 0.5% deoxycholic acid, and 0.1% SDS, and the concentration of protein was determined by bicinchoninic acid (BCA) assay. Cell lysates (30 μg protein) were separated by SDS-PAGE and blotted onto PVDF membranes. After blocking in blocking buffer containing 0.05% Tween 20 and 5% skim-milk in 50 mM Tris-HCl for 30 min, the blots were incubated with primary antibodies (S100β and β-actin) overnight. The membrane was washed and then treated with HRP-conjugated secondary antibodies for 1 h. The immune-reactive bands were analyzed by using enhanced chemiluminescence solution (Thermo Scientific).

### Preparation of iPSC-derived Schwann cells (iPSC-SCs) and primary Schwann cells

iPSCs derived from human newborn foreskin fibroblasts (catalog number CRL-2097; ATCC) were cultured and iPSC-SCs were prepared as described previously [25]. Primary human Schwann cells were purchased from ScienCell, and they were cultured in Schwann cell growth medium (ScienCell).

### Enzyme-linked immunosorbent assay (ELISA)

A total of 1 × 10^5^ iSCPs, iSCs, iPSC-SCs, or pSCs in 0.5 ml were seeded in 24-well culture. After 24 h, the cultured media were filtered through a 0.22-μm filter (Millipore). To measure the concentration of secreted neurotrophic factors (BDNF, GDNF, β-NGF, and NT-3), ELISAs were performed on conditioned medium derived from iSCPs and iSCP-SCs according to the manufacturer’s protocol (Abcam, Cambridge, MA).

### DRG neuron co-culture with iSCs in microfluidic chamber

DRG neurons were prepared from embryonic day 15 rat pups as previously described [25]. Rat DRG neurons were plated in DRG growth medium containing 4 g/L d-glucose (Sigma), 50 ng/ml NGF (R&D), and 15% FBS (Thermo Scientific) in MEM (Thermo Scientific) onto 12 mm poly-d-lysine and laminin-coated microfluidic chamber (Millipore, AX45005PBC). To eliminate endogenous nonneuronal cells, the cultures were treated with 100 nM Ara-C (Sigma), 4 g/L d-glucose (Sigma), 50 ng/ml NGF (R&D Systems), 1% FBS (Thermo Scientific), and 1 × B27 (Thermo Scientific) in neurobasal medium for 3 days and then changed into DRG growth medium for 1 day on the seventh culture day. DRGs were then maintained in DRG differentiation medium containing 4 g/L d-glucose, 50 ng/ml NGF ((R&D Systems), 1% FBS (Thermo Scientific), and 1 × B27 (Thermo Scientific) in neurobasal medium before coculture with SCs. A total of 2 × 10^3^ SCs were added to the axonal side of the microfluidic chamber in DRG SC medium for 7 days. Bright field images were obtained by using an Axio VertA.1 microscope (Germany).

### In vitro myelination with human sensory neurons (HSNs)

Human iPSCs (hiPSCs) from CRL-2097 [25] were maintained on Matrigel-coated plates with mTeSR™1 medium (Stemcell Technologies, Vancouver, BC, Canada). The hiPSCs were directly differentiated into sensory neurons as previously described [26, 27]. For HSN differentiation, hiPSCs were incubated in knockout medium (KSR medium) containing 15% knockout-serum replacement (ThermoFisher), 1% Glutamax I (ThermoFisher), 1% nonessential amino acids (ThermoFisher), 100 μM β-mercaptoethanol, 1% antibiotic/antimycotic (ThermoFisher), 10 μM SB431542 (Sigma), and 100 nM LDN-193189 (Stemgent). The culture medium was gradually switched from KSR medium to N2 medium containing 2% B27 supplement, 1% N2 supplement, 1% Glutamax I, and 1% antibiotic/antimycotic in neurobasal medium (25% N2 medium on day 4, 50% N2 medium on day 6, 75% N2 medium on day 8, and 100% N2 medium on day 10). Three small molecule inhibitors, 3 μM CHIR99021 (Tocris), 10 μM SU5402 (Tocris), and 10 μM DAPT (Sigma), were added to the culture medium on day 2 through day 10. On day 6, the SMAD inhibitors (SB431542 and LDN-193189) were removed, leaving only three inhibitors in the medium. On day 10, the cells were replated using accutase (Millipore) onto Matrigel-coated 24-well plates (30,000 *per* well) in N2 medium containing human recombinant 20 ng/ml NGF (Peprotech), 20 ng/ml GDNF (Peprotech), 20 ng/ml BDNF (Peprotech), and 20 ng/ml NT3 (Peprotech). CHIR99021 (3 μM) was added to the medium until day 15. On day 18, the cells were treated with 100 nM Ara-C (Sigma) for 24 h to remove nonneuronal cells. A total of 15,000 or 20,000 iSCs were added to the HSN cultures in coculture medium containing 1% N2 supplement, 0.2% FBS, 1% Glutamax I, and 1% antibiotic/antimycotic in DMEM/F12 medium and maintained for 7 days to allow neuritogenesis. Myelination was induced with myelination medium containing 1% N2 supplement, 1% FBS 1%, Glutamax I, 1% antibiotic/antimycotic, and 50 ng/ml ascorbic acid (Sigma) in DMEM/F12 medium for 3 weeks. The medium was refreshed every day.

### Sciatic nerve surgical procedure and cell transplantation

The surgical procedure used to establish sciatic nerve injury and cell transplantation was performed as previously described [25]. Briefly, 8-week-old C57BL/6 male mice were anesthetized, and the left sciatic nerve was cut approximately 2–3 mm. The prepared iSCs or iPSC-SCs were then transplanted into a 2–3-mm gap in the transected sciatic nerve of mice. The cell transplantation group was divided into two groups as follows: group I, Matrigel (5 μl)-treated mice considered the control, and group II, Matrigel- and iSC (1 × 10^5^ cells/5 μl)-treated mice. Data analysis was performed 4 and 8 weeks after surgery for each group.

### Rotarod test

The recovery of nerve function in sciatic nerve-injured mice was evaluated by a rotarod test (Daejong Instrument Industry, South Korea) after cell transplantation. All mouse groups received a preoperative performance evaluation at 7 to 8 weeks of age. Control and iSC-transplanted mice were placed on an accelerating rotarod that was programmed to accelerate from 4 to 40 rpm in 180 s and maintained at a constant speed for 120 s. Then, the latency to fall was recorded. Three trials per test were administered to the mice during the test day, with a 10-min intertrial interval between trials. The mean latency of three trials was considered for analysis.

### Analysis of gastrocnemius muscles

For the analysis of gastrocnemius muscles, the control and iSC-transplanted areas were excised. To calculate the gastrocnemius muscle volume, the length × width × height of the sample was measured using a Digimatic Caliper (Mitutoyo). The formula was [(length × width × height)/2] (mm^3^). The wet weights of control and iSC-injected gastrocnemius muscle were measured, and the ratios were compared. Gastrocnemius muscle samples were fixed in 4% paraformaldehyde for cross-sectional area (CSA) analysis. After sectioning and dehydration, Masson’s trichrome (#SSK5005-250, BBC Chemical) staining was performed according to the manufacturer’s instructions. Analysis was performed with ImageJ software.

### Statistical analysis

The results are presented as the mean ± S.E.M. Student’s unpaired *t* test was used for statistical evaluation, with *p* values of 0.05, 0.01, or 0.001 as the level of significance.

### SCPs can be directly reprogrammed from human fibroblasts

**Fig. 1:**
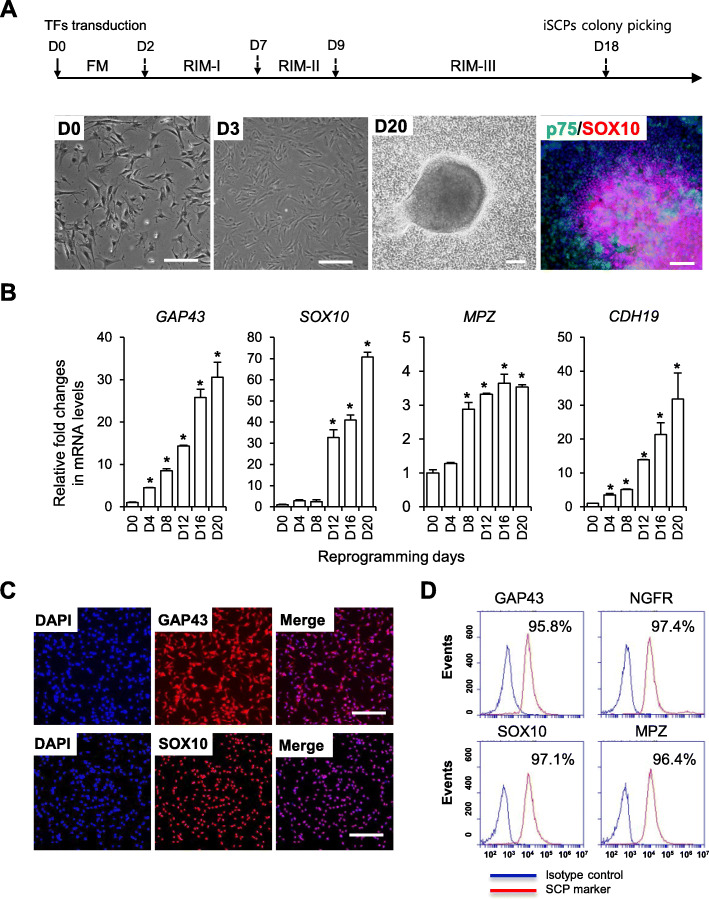
Generation of iSCPs form human fibroblasts. **a** Schematic diagram of the iSCP reprogramming process (top). Bright-field images of reprogramming cells (bottom). The iSCP colony (day 18) was coimmunostainned with SCP markers (p75 and SOX10) (bottom). Scale bars = 100 μm. **b** qPCR analysis of SCP markers (*GAP43*, *SOX10*, *MPZ*, and *CDH19*) during reprogramming. All the values are relative to human fibroblasts. Mean ± S.E.M. (*n* = 3–5). **c** Immunocytochemical analysis of SCP markers (SOX10 and GAP43) in iSCPs (day 18). Scale bars, 100 μm. **d** Flow cytometry analysis of SCP markers (GAP43, NGFR, SOX10, and MPZ) in iSCPs (day 18). Isotype controls are indicated in blue

**Fig. 2:**
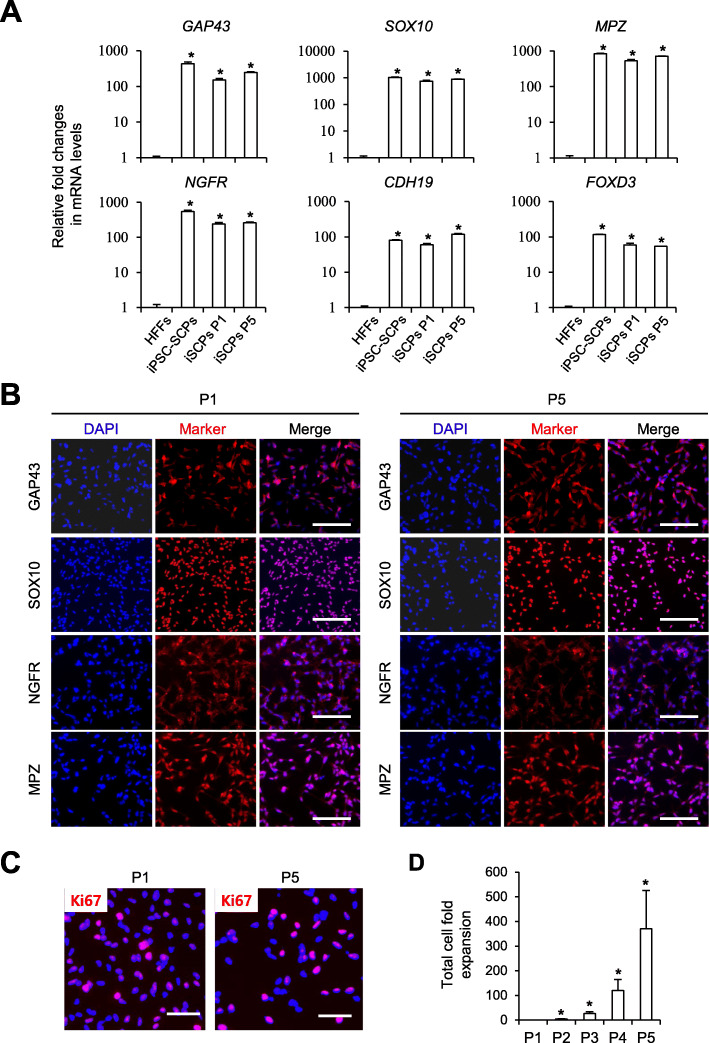
Propagation of iSCP in vitro. **a** qPCR analysis of SCP markers (*GAP43*, *SOX10*, *MPZ*, *NGFR*, *FOXD3*, and *CDH19*) in human fibroblasts (HFFs), and hiPSC-differentiated SCPs (iPSC-SCPs), passage 1 (P1), and passage (P5). **b** Immunocytochemical analysis of SCP markers (GAP43, SOX10, NGFR, and MPZ) in SCPs at P1 and P5. **c** Immunocytochemical analysis of the proliferation marker Ki67 (red) in iSCPs at P1 and P5. Scale bars, 100 μm. **d** The fold expansion of the iSCPs at the indicated passages relative to passage 1. The cell numbers were counted with the use of a hemocytometer. Mean ± S.E.M. (*n* = 6)

Previously, we defined an SCP lineage-specific medium condition for hPSC differentiation into SCPs [25]. With a modified stepwise use of SCP-reprogramming induction medium (RIM-I/II/III), integration-free induced SCPs (iSCPs) were successfully converted from human fibroblasts by introducing oriP/EBNA-based episomal vectors encoding the pluripotency core factors *OCT4*, *SOX2*, *KLF4*, *MYCL1*, and *LIN28* and p53 shRNA (Fig. 1a). Reprogramming factor gene expression picked at day 8 post-transfection, then declined toward baseline levels comparable to untransfected starting cells (Fig. S1A). ISCPs morphologically formed colonies, similar to hPSC-derived SCPs, during reprogramming culture and could be isolated for further analyses and processes on day 18 (Fig. 1a). The identity of iSCPs was phenotypically confirmed by lineage-specific expression of *GAP43, SOX10*, *MPZ*, and *CDH19* at the RNA level (Fig. 1b) and GAP43, SOX10, NGFR, and MPZ at the protein level (Fig. 1c, d). More than 95% of reprogrammed cells were positive for all tested SCP marker proteins (Fig. 1d). Total expression levels of *OCT4*, *SOX2*, *KLF4*, and *MYCL1* in established iSCPs were significantly lower than those seen in the same episomal vector-mediated iPSCs (Fig. S1A). The quantitative RT-PCR analysis has also confirmed the absence of exogenous reprogramming factor transgenes in established iSCPs (Fig. S1B). Established iSCPs could be dissociated with accutase enzyme treatment without a significant loss of viability and further stably expanded in vitro over 3 weeks without morphological changes and without a loss of SCP marker expression (Fig. 2a, b) and proliferation capacity (Fig. 2c, d). Passaged iSCPs (p5) maintained similar expression levels of SCP marker genes (*GAP43*, *SOX10*, *MPZ*, *NGFR*, *CDH19*, and *FOXD3*) (Fig. 2a) and proteins (GAP43, SOX10, MPZ, and NGFR) (Fig. 2b). The distribution of proliferating Ki-67-positive cells remained similar between passages (P1: 71.4 ± 5.6%, P5: 70.4 ± 4.3%) (Fig. 2c). Our results demonstrate that highly pure, renewable SCPs can be directly converted from human fibroblasts with pluripotency TFs in combination with optimized SCP induction medium.

### ISCPs are capable of differentiating into SCs

**Fig. 3:**
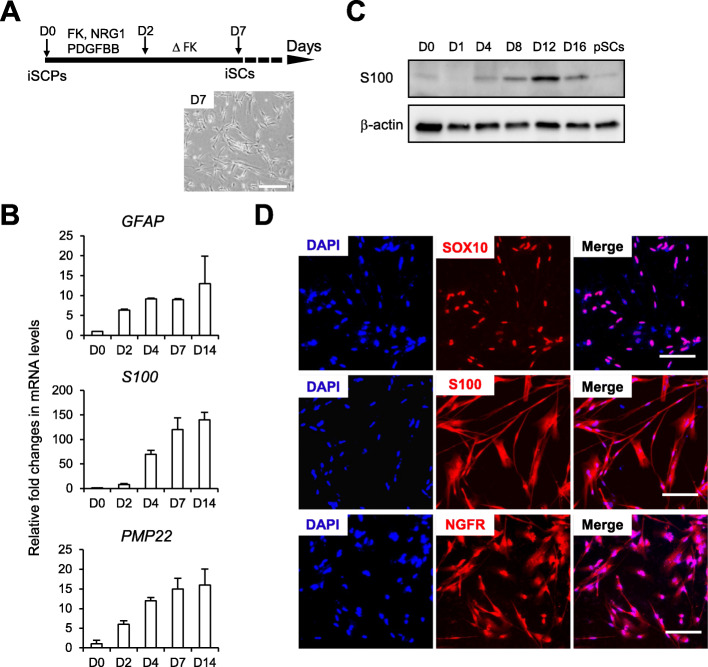
Differentiation of iSCPs into Schwann cells. **a** Schematic diagram of the iSC differentiation process (top). Representative phase-contrast images of differentiated iSCs (day 7). Scale bars = 200 μm. **b** qPCR analysis of SC markers (*GFAP*, *S100b*, and *PMP22*) during differentiation. All the values are relative to day 0 of differentiation. Mean ± S.E.M. (*n* = 3–5). **c** Immunoblot analysis of the SC marker S100B during differentiation. Whole cell lysates were electrophoresed by SDS-PAGE, and primary SCs (pSCs) were used as a control. **d** Immunostaining for the SC markers (SOX10, S100B, and NGFR) after 7 days of differentiation. Cell nuclei were stained with DAPI (blue). Scale bars = 100 μm

We further investigated whether iSCPs can be induced to differentiate into SCs. Most iSCPs were effectively differentiated into spindle-like SCs, similar to hPSC-SCP-derived SCs, at approximately 7 days postdifferentiation by employing previously reported SC differentiation medium containing forskolin and a high concentration of NRG1β (200 ng/ml) (Fig. 3a) [25]. The identity of iSCP-differentiated SCs (iSCs) was verified by the lineage-specific expression of *GFAP*, *S100b*, and *PMP22* at the RNA level (Fig. 3b), and SOX10, S100b, and MPZ at the protein level (Fig. 3c, d). More than 95% of differentiated cells were positive for all tested SC phenotypic marker proteins (Fig. 3d) after 1 week of differentiation. In addition, the levels of BDNF, GDNF, NGF, and NT-3, known as crucial factors for axonal regeneration in the PNS, were significantly increased in iSC-cultured medium compared to undifferentiated iSCP-cultured control medium, as confirmed by ELISA (Fig. S2), indicating the secretion of neurotropic factors in iSCs. Notably, iSCs secreted greater amounts of BDNF and GDNF than positive control cells, primary SCs (pSCs) and iPSC-differentiated SCs (iPSC-SCs) (Fig. S2). NGF- and NT-3-secreted levels from iSCs were slightly higher or similar compared to those from pSCs and iPSC-SCs (Fig. S2). Our results show that iSCPs are a useful source for SC production.

### ISCs are functionally effective in promoting axon outgrowth and myelination in vitro

To determine whether iSCs have the ability to promote axonal growth and myelination in vitro, we used two coculture models: an iSC and rat DRG neuron model and an iSC and hiPSC-derived sensory neuron (HSN) model. In the microfluidic chamber coculture system, axon length was increased, and the numbers of axons crossing the microgrooves were markedly increased in rat DRG neurons cocultured with iSCs compared with monocultured DRG neurons (Fig. S3), suggesting the axon growth-promoting activity of iSCs.

**Fig. 4:**
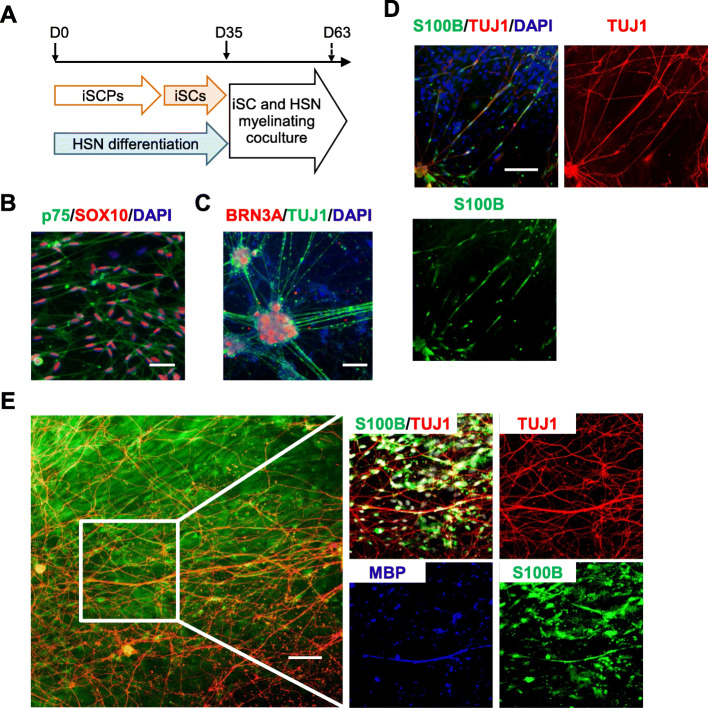
Coculture of iSCs with HSNs. **a** Schematic diagram of the coculture protocol of iSCs and HSNs. **b** The iSCs differentiated from iSCPs were immunostained for SOX10 (red), p75 (green), and human nucleus (blue). **c** HSNs differentiated from iPSCs were immunostained for BRN3A (red), TUJ1 (green), and human nucleus (blue). **d** The iSCs cocultured with HSNs for 14 days were immunostained for TUJ1 (red) and S100B (green). **e** iSCs cocultured with HSNs for 28 days were immunostained for MBP (blue), TUJ1 (red), and S100B (green) (left). High magnification of the boxed area (right). Scale bars = 100 μm

The iSCs were further assessed for myelination function in coculture with HSNs (Fig. 4a). Prepared iSCs (Fig. 4c) were added to established HSN culture (Fig. 4b) and allowed to myelinate. By day 14 in mixed coculture, the iSCs were closely associated with and aligned along outgrowing neurites of HSNs, as visualized with immunostaining for S100b and neuronal-specific tubulin (TUJ-1) (Fig. 4d). In addition, myelin basic protein (MBP)-positive iSC myelin segments (12.6 ± 8.1 myelin segments *per* well of a 24 well plate, *n* = 8) were observed along neurite segments at day 28 of mixed culture (Fig. 4e). These results clearly demonstrate that iSCs have the capacity to promote axon outgrowth and myelination in vitro, in part, by secreting neurotrophic factors such as BDNF, GDNF, NGF, and NT-3.

### ISCs have the ability to improve sciatic nerve regeneration in vivo

**Fig. 5:**
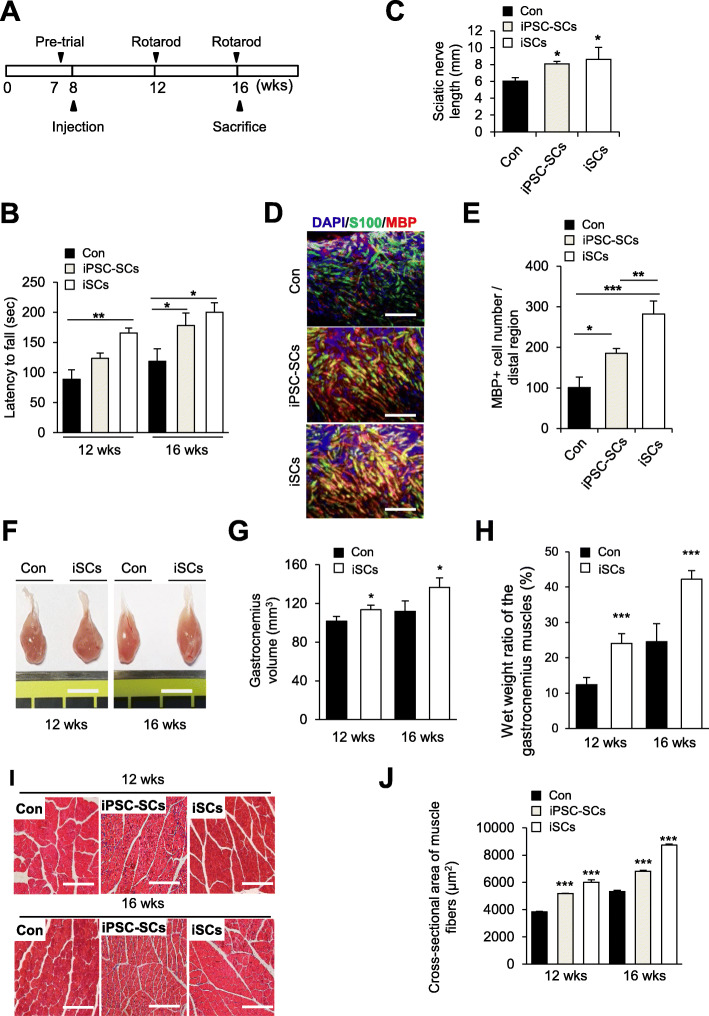
Effects of transplanted iSCs on a sciatic nerve injury model in vivo. **a** The rotarod tests consisted of an accelerated rotarod phase (4 rpm to 40 rpm/3 min) and a fixed speed rotarod phase (40 rpm/2 min) and were conducted at four-weeks intervals. **b** Three tests were performed per test on the day of the test, with an interval of 10 min per test. The time to fall off the rotarod was recorded in Matrigel-treated groups (Con), iPSC-SC-treated groups (iPSC-SCs), and iSC-treated groups (iSCs). **c** The length of the resected sciatic nerve was measured using a caliper at 16 weeks after injury. Mean ± S.E.M. (*n* = 6 mice per group). **d** Eight weeks later the regenerated sciatic nerve region was excised and immunostained with anti-S100 (green) and anti-MBP (red) antibodies. Scale bars, 200 μm. **e** Evaluation of iSC-transplanted gastrocnemius muscle atrophy. **f** Macroscopic views of gastrocnemius muscles at 12 and 16 weeks after surgery. Scale bars, 1 cm. **g** The volume of gastrocnemius muscles at 12 and 16 weeks after surgery. **h** Quantitative analysis of weight and wet weight ratios of gastrocnemius muscles at 12 and 16 weeks after surgery. **i** Masson’s trichrome staining of cross-sections of gastrocnemius muscles at 12 and 16 weeks after surgery. Scale bars: 100 μm **j** The mean cross-sectional area (CSA) of gastrocnemius muscle fibers in the control and iSC-transplanted groups (*n* = 5). *p < 0 .05, **p < 0.01, ***p < 0.001. Control, no-cell transplanted mouse group; iPSC-SC, iPSC-SC-transplanted mouse group; iSC, iSC-transplanted mouse group

To evaluate the capacity of iSCs for axonal regeneration in the PNS in vivo, iSCs and iPSC-SCs (as a positive control) were transplanted into a sciatic nerve injury mouse model. The rotarod test was performed to assess the functional recovery of motor coordination and balance after iSC transplantation. The latencies of mice to fall off the rotating rod were significantly improved in the iSC-transplanted groups compared to the control groups (Fig. 5a). At 12 weeks, the control group injected with Matrigel (*n* = 6) averaged 89 s, whereas the iSC- and iPSC-SCs injected group (*n* = 6) had an average of 165 and 123 s, respectively (Fig. 5b). At 16 weeks, the control group averaged 118 s and the iSC- and iPSC-SCs injected group averaged 200 and 178 s, respectively (Fig. 5b).

Improved sciatic nerve regeneration from the proximal stump to the distal stump was observed at 16 weeks in iSC-transplanted groups, whereas almost no regeneration was observed in the Matrigel-treated control groups (Fig. 5c–e). The length of the resected sciatic nerve was significantly increased in the iSC-transplanted groups (8.62 ± 1.4 mm) compared to the control groups (6.04 ± 0.4 mm) (Fig. 5c). The number of S100-positive and MBP-positive myelinated fibers was also significantly increased in iSC-transplanted groups compared to the Matrigel-treated and iPSC-SC-treated groups (Fig. 5d, e).

We also investigated whether newly regenerated axons can reinnervate gastrocnemius muscles, one of main target muscles of the sciatic nerve. As a result, the volume of gastrocnemius muscle was significantly increased in the iSC-transplanted groups (136.5 ± 15.3 mm^3^) compared to the Matrigel-treated control groups (111.7 ± 10.8 mm^3^) (Fig. 5f, g). The wet weight of gastrocnemius muscle in the iSC-transplanted groups (at 12 weeks: 24.0 ± 2.7%, at 16 weeks: 42.2 ± 2.4%) was also significantly increased compared to that in the control groups (at 12 weeks, 12.3 ± 2.0%; at 16 weeks, 24.5 ± 5.1%) (Fig. 5h). We also found that iSC transplantation enhanced the CASA of gastrocnemius muscle fibers by 1.63-fold in iSC-transplanted mice compared to control mice (Matrigel-treated groups at 12 weeks: 3844.8 ± 29.2 μm^2^, at 16 weeks: 5331.0 ± 86.0 μm^2^; iPSC-SC-treated groups at 12 weeks: 5188.0 ± 9.5 μm^2^, at 16 weeks: 6825.5 ± 62.2 μm^2^; iSC at 12 weeks: 6005.2 ± 190.2 μm^2^, at 16 weeks: 8733.0 ± 75.3 μm^2^). Taken together, our results suggest that transplantation of iSCs can enhance peripheral nerve regeneration and functional recovery.

## Discussion

Previously, we developed a differentiation strategy to produce SCPs from hPSCs [25]. With another approach, here, we demonstrate for the first time the generation of integration-free, functional, and expandable SCPs from human fibroblasts. Direct lineage conversion to a specific cell type can be achieved by modifying the iPSC reprogramming process. Studies demonstrate that the transient expression of iPSC reprogramming TF genes can be used as an alternative to lineage-specific TFs to direct various specific lineages [28–32]. We generated iSCPs with iPSC TF-expressing integration-free episomal vectors. Treating the iPSC TF-induced intermediate cells (reprogramming days 6–8) with developmental cues (i.e., SB431542, CT99021, and NRG1) resulted in the significant induction of high purity SCPs (over 95%) from human fibroblasts (Fig. 1b). No significant expression of pluripotency markers, such as NANOG and SSEA4, was observed during the reprogramming process. Using a direct conversion strategy avoiding the pluripotent state, we can limit the potential risk of tumor/teratoma formation arising from the use of hPSC derivatives and expand the choice of target donor cells that may bring iSCPs closer to clinical use.

We confirmed that the iSCPs were phenotypically and functionally similar to hPSC-SCPs [25]. They express cell markers (GAP43, SOX10, MPZ, CDH19, NGFR, and FOXD3) that are typical for SCPs and have the ability to expand and differentiate into high purity SCs (more over 95%) using the same differentiation medium used for hPSC-SCPs in vitro. ISCs from iSCPs effectively promoted axonal growth and myelination both in vitro and in vivo. After coculture with rat DRG neurons in a microfluidic chamber system, iSCs significantly enhanced axonal outgrowth of DRG neurons. In addition, coculture of iSCs with human sensory neurons led to enhanced myelination, as revealed by MBP-positive myelin segments. Transplanted iSCs effectively integrated into regenerating sciatic nerve tissue with MBP expression. The myelination efficacy and functional relevance of iSCs were analogous to those of hPSC-SCs, so iSCs might be another suitable source for SC therapy for peripheral nerve injury.

Heterogamous peripheral glial cells including myelinating and nonmyelinating SCs and other specialized subtypes of glia are known to originate from neural-crest-derived SCPs [33, 34]. Despite extensive studies, the understanding of the precise mechanisms driving peripheral glial/SC specification and PNS myelination and the similarity and difference between SCPs and oligodendrocyte precursor cells, which produce myelinating cells in the central nervous system, are still largely limited. Thus, acquiring novel intermediate stage cell types for PNS development, especially at the SCP stage, is an important issue to overcome the limitations of proper cell sources. Currently, there are various options for obtaining functional human SCs using differentiation strategies based on the NCC intermediate stage [4, 13, 16] or direct reprogramming strategies without conversion to the pluripotency stage [20, 22, 23, 29], but no strategies are available for SCP intermediate production. Our iSCP strategy addresses the limitations of SC lineage cell sources and provides an alternative way to obtain functional SCPs/SCs to investigate the molecular mechanisms underlying SC lineage conversion and differentiation.

## Conclusions

In summary, we report a simple direct reprogramming strategy for the generation of human Schwann cell precursors (SCPs) as a promising source for functional SCs. Our iSCP strategy is also valuable for the generation of patient- and disease-specific SCP/SCs, which could be a promising tool for the in vitro modeling of PNS diseases, drug discovery, and clinical studies.

## Supplementary information

**Additional file 1: Figure S1.** qPCR analysis of pluripotent factor in iSCPs. **Figure S2.** Secretion levels of neurotrophic factors from cultured iSCPs, iPSC-SCs, iSCs, and pSCs. **Figure S3.** Coculture of iSCs and rat DRG neurons in microfluidic chamber.

**Additional file 2: Table S1.** Primers for qRT-PCR. **Table S2.** List of antibodies.

## Abbreviations

iSCPs: Induced-Schwann cell precursors
iSCs: iSCP-derived Schwann cells
iPSC: Induced pluripotent stem cells
hESCs: Human embryonic stem cells
NCSCs: Neural crest stem cells
hPSCs: Human pluripotent stem cells
SCPs: Schwann cell precursors
RIM: Reprogramming induction medium
cDNA: Complementary DNA
DAPI: 4′,6-Diamidino-2-phenylindole
FBS: Fetal bovine serum
qRT-PCR: Quantitative reverse transcription polymerase chain reaction
GAPDH: Glyceraldehyde 3-phosphate dehydrogenase
pSC: Primary human Schwann cell
ELISA: Enzyme-linked immunosorbent assay
GDNF: Glial cell line-derived neurotrophic factor
BDNF: Brain-derived neurotrophic factor
NGF: Nerve growth factor
NT-3: Neurotrophin-3
GFAP: Glial fibrillary acidic protein
HRP: Horseradish peroxidase
NRG1: Recombinant human heregulin-β1
NGFR: Nerve growth factor receptor p75
MPZ: Myelin protein zero
PBS: Phosphate-buffered saline
PDGF: Platelet-derived growth factor
DRG: Dorsal root ganglion
PVDF: Polyvinylidene difluoride membrane
SDS-PAGE: Sodium dodecyl sulfate-polyacrylamide gel electrophoresis
