# Supplementary figures and images for: Directly induced human Schwann cell precursors as a valuable source of Schwann cells
Source: Stem Cell Res Ther. 2020 Jun 26;11:257. doi: 10.1186/s13287-020-01772-x (PMC7318441; doi:10.1186/s13287-020-01772-x)

# Figure S1

**A**

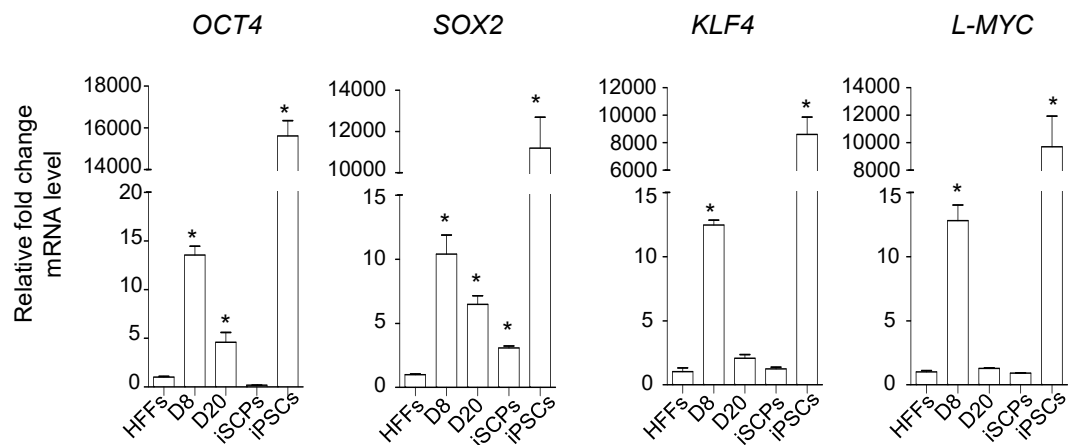

**B**

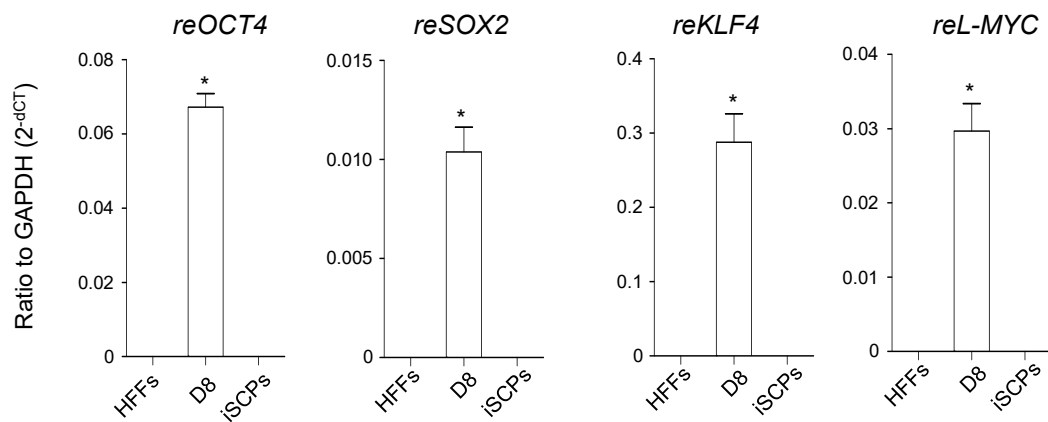

# Figure S2

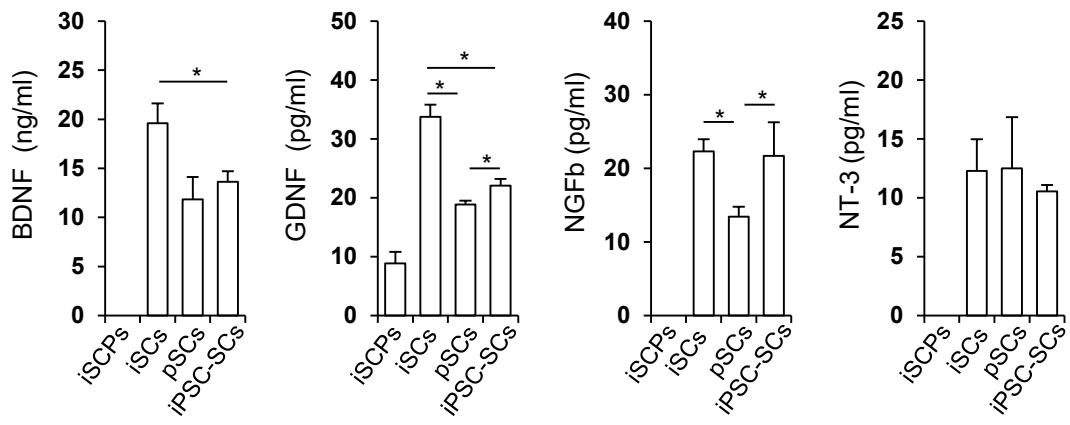

# Figure S3

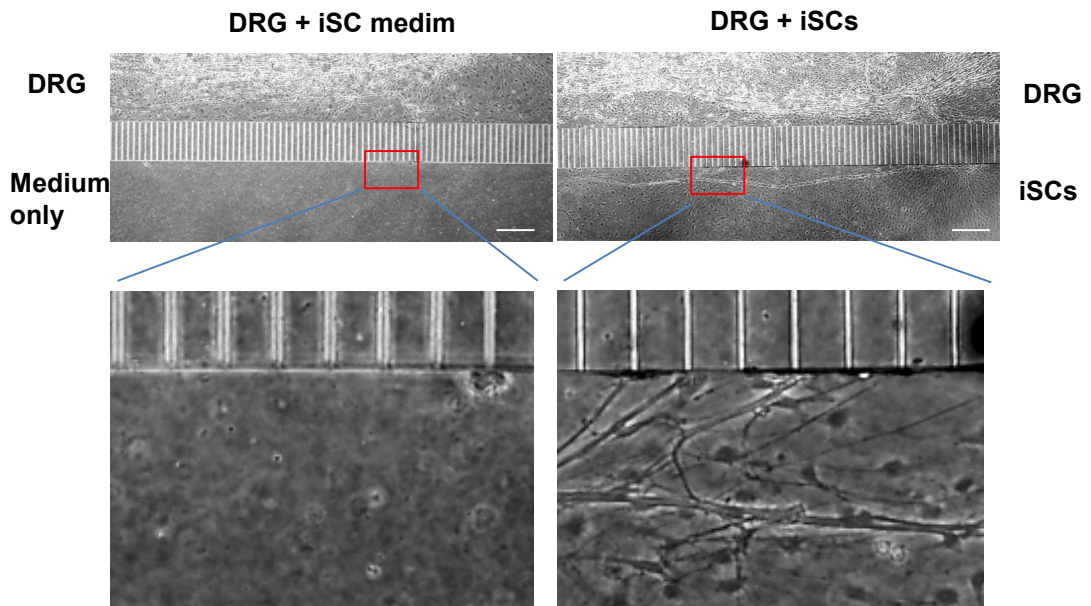

Supplement: Supplementary file 1 — Additional file 1: Figure S1. qPCR analysis of pluripotent factor in iSCPs. Figure S2. Secretion levels of neurotrophic factors from cultured iSCPs, iPSC-SCs, iSCs, and pSCs. Figure S3. Coculture of iSCs and rat DRG neurons in microfluidic chamber. [file 13287_2020_1772_MOESM1_ESM.pdf]
