# Supplementary material for: Directly induced human Schwann cell precursors as a valuable source of Schwann cells
Source: Stem Cell Res Ther. 2020 Jun 26;11:257. doi: 10.1186/s13287-020-01772-x (PMC7318441; doi:10.1186/s13287-020-01772-x)
Supplement: Supplementary file 2 — Additional file 2: Table S1. Primers for qRT-PCR. Table S2. List of antibodies. [file 13287_2020_1772_MOESM2_ESM.docx]

**Supplemental Information**

**Directly induced human Schwann cell precursors as a valuable source of Schwann cells**

Han-Seop Kim^1,§^, Jae Yun Kim^1,2,§^, Cho Lok Song^1,2^, Ji En Jeong^1^, and Yee Sook Cho^1,2*^

^1^Stem Cell Research Laboratory, Immunotherapy Research Center (IRC), Korea Research Institute of Bioscience and Biotechnology (KRIBB), 125 Gwahak-ro, Yuseong-gu, Daejeon,^2^Department of Bioscience, KRIBB School, University of Science & Technology, 113 Gwahak-ro, Yuseong-gu, Daejeon 34113, South Korea

**Figure S1. qPCR analysis of pluripotent factor in iSCPs. (**A) qPCR analysis of reprogramming factors (OCT4, SOX2, KLF4, and L-Myc) in human fibroblasts (HFFs), cells at different days post-reprogramming [day 8 (D8) and day 20 (D20)], established iSCPs (passage 3), and HFF-derived episomal iPSC line (iPSCs). (B) Residual reprogramming factor expression in indicated samples. Passage 5 iPSCs were used as a positive control. Mean ± S.E.M. (n=3). ∗p<0.01 (vs HFF in A) using a t-test.

**Figure S2. Secretion levels of neurotrophic factors from cultured iSCPs, iPSC-SCs, iSCs, and pSCs**. Mean concentrations of neurotrophic factors (BDNF, GDNF, NGF, and NT3) secreted in the culture supernatants of iSCPs, iSCs, iPSC-differentiated SCs (iPSC-SCs), and primary SCs (pSCs) were measured with ELISA. Mean ± S.E.M. (n=4). ∗p<0.05 using a t-test. ns; no significance.

**Figure S3. Coculture of iSCs and rat DRG neurons in microfluidic chamber**. Rat DRG neurons were cultured with or without iSCs for 14 days in a microfluidic chamber, and axonal growth through the microchannel was analyzed by microscopy. Scale bars = 450 μm.

**Table S1.** Primers for qRT-PCR

| **Gene** | **Sequence (5'-->3')** | |
| --- | --- | --- |
| *SOX10* | Forward | *CCT CAC AGA TCG CCT ACA CC* |
|  | Reverse | *CAT ATA GGA GAA GGC CGA GTA GA* |
| *FOXD3* | Forward | *GAC GCA GGT TGC GAT AGC C* |
|  | Reverse | *CGC CTC CTT GGG CAA TGT C* |
| *MPZ* | Forward | *AAG TGC CAA CTA GGT ACG GG* |
|  | Reverse | *CAT AGC ACT GAG CCT CCT CT* |
| *CDH19* | Forward | *ACA AGC GTC TGT AAC TCT GGG* |
|  | Reverse | *AGC AAA CTT CGT GTT GGA CA* |
| *NGFR* | Forward | *TGG CCT ACA TAG CCT TCA AGA* |
|  | Reverse | *GAG ATG CCA CTG TCG CTG T* |
| *GAP43* | Forward | *GGC CGC AAC CAA AAT TCA GG* |
|  | Reverse | *CGG CAG TAG TGG TGC CTT C* |
| *GAPDH* | Forward | *ACA ACT TTG GTA TCG TGG AAG G* |
|  | Reverse | *GCC ATC ACG CCA CAG TTT C* |
| *S100b* | Forward | *GAC CCT CAT CAA CGT GTT CCA* |
|  | Reverse | *CCA CAA GCA CCA CAT ACT CCT* |
| *GFAP* | Forward | *AGG TCC ATG TGG AGC TTG AC* |
|  | Reverse | *GCC ATT GCC TCA TAC TGC GT* |
| *PMP22* | Forward | *GAT CCT GTC GAT CAT CTT CAG C* |
|  | Reverse | *AGC ACT CAT CAC GCA CAG AC* |
| *OCT4* | Forward | *GAG AAG GAT GTG GTC CGA GTG TG* |
|  | Reverse | *CAG AGG AAA GGA CAC TGG TCC C* |
| *SOX2* | Forward | *AGA ACC CCA AGA TGC ACA AC* |
|  | Reverse | *ATG TAG GTC TGC GAG CTG GT* |
| *KLF4* | Forward | *GTC TCT TCG TGC ACC CAC TT* |
|  | Reverse | *AAG AAG GTG GGG TGA GCA TC* |
| *L-Myc* | Forward | *ATG GAC TAC GAC TCG TAC CAG CAC* |
|  | Reverse | *CGT ATG ATG GAG GCG TAG TTC CTG* |
| *reOCT4* | Forward | *CAT TCA AAC TGA GGT AAG GG* |
|  | Reverse | *TAG CGT AAA AGG AGC AAC ATA G* |
| *reSOX2* | Forward | *TTC ACA TGT CCC AGC ACT ACC AGA* |
|  | Reverse | *TTT GTT TGA CAG GAG CGA CAA T* |
| *reKLF4* | Forward | *CCA CCT CGC CTT ACA CAT GAA GA* |
|  | Reverse | *TAG CGT AAA AGG AGC AAC ATA G* |
| *reL-Myc* | Forward | *GGC TGA GAA GAG GAT GGC TAC* |
|  | Reverse | *TTT GTT TGA CAG GAG CGA CAA T* |

**Table S2. List of antibodies**

| **Protein** | **Company (Cat. No.)** | **Dilution** |
| --- | --- | --- |
| SOX10 | Abcam (AB155279) | 1:200 |
| SOX10 | Bioss (bs-6449R-A488) | 1:200 |
| NGFR | Abcam (AB3125) | 1:50 |
| NGFR | Bioss (bs-0161R-A647) | 1:200 |
| TUJ-1 | Covance (MRB-435P) | 1:1000 |
| GAP43 | Abcam (AB75810) | 1:500 |
| GAP43 | Bioss (bs-0154R-A647) | 1:100 |
| MPZ | Abcam (AB31851) | 1:100 |
| MPZ | Abcam (AB39375) | 1:100 |
| MPZ | Bioss (bs-0337R-A488) | 1:100 |
| MBP | Millipore (MAB386) | 1:300 |
| S100b | Abcam (AB52642) | 1:200 |
| Ki67 | BD (550609) | 1:50 |
